# Supplementary material for: Deletion of wfs1 Impairs Oligodendrocyte Precursor Cells Dorsal Distribution and Myelination Through the wfs1-hmgcs1 Axis in Zebrafish
Source: Biology (Basel). 2026 Aug 16;15(16):1402. doi: 10.3390/biology15161402 (PMC13510151; doi:10.3390/biology15161402)
Supplement: Supplementary file 1 [file biology-15-01402-s001.zip › File S1-Transcriptome sequencing data and differentially expressed genes (DEGs) in wfs1-- zebrafish at 5 dpf/2.KEGG/wfs1KOvsWT/GSEA/wfs1KO/TRYPTOPHAN_METABOLISM/TRYPTOPHAN_METABOLISM(DRE00380).html]

Details for gene set TRYPTOPHAN\_METABOLISM(DRE00380)[GSEA]

|  || Dataset | deg.sample |
| Phenotype | sample.cls |
| Upregulated in class | wfs1KO |
| GeneSet | TRYPTOPHAN\_METABOLISM(DRE00380) |
| Enrichment Score (ES) | 0.31294072 |
| Normalized Enrichment Score (NES) | 1.3403127 |
| Nominal p-value | 0.08730159 |
| FDR q-value | 0.2686199 |
| FWER p-Value | 0.931 |
Table: GSEA Results Summary

  

Fig 1: Enrichment plot: TRYPTOPHAN\_METABOLISM(DRE00380)      
 Profile of the Running ES Score & Positions of GeneSet Members on the Rank Ordered List

  

| PROBE | DESCRIPTION (from dataset) | GENE SYMBOL | GENE\_TITLE | RANK IN GENE LIST | RANK METRIC SCORE | RUNNING ES | CORE ENRICHMENT || 1 | 447801\_1 | aldh8a1 |  |  | 45 | 4.909 | 0.0707 | Yes |
| 2 | 103911233 | LOC103911233 |  |  | 562 | 2.827 | 0.0961 | Yes |
| 3 | 103911241 | LOC103911241 |  |  | 1022 | 2.074 | 0.1121 | Yes |
| 4 | 393860 | gcdha |  |  | 1119 | 1.976 | 0.1382 | Yes |
| 5 | 571967 | ido1 |  |  | 1123 | 1.972 | 0.1670 | Yes |
| 6 | 368239 | aldh2.2 |  |  | 1221 | 1.881 | 0.1916 | Yes |
| 7 | 555401 | aoc1 |  |  | 1405 | 1.721 | 0.2112 | Yes |
| 8 | 103911886 | si:dkey-93h22.4 |  |  | 1514 | 1.645 | 0.2320 | Yes |
| 9 | 553401 | hadhaa |  |  | 1608 | 1.593 | 0.2525 | Yes |
| 10 | 447920 | aldh3a2b |  |  | 1830 | 1.444 | 0.2667 | Yes |
| 11 | 100334830 | kynu |  |  | 2298 | 1.249 | 0.2704 | Yes |
| 12 | 393677 | aanat1 |  |  | 2622 | 1.139 | 0.2770 | Yes |
| 13 | 368262\_1 | dlst |  |  | 2921 | 1.056 | 0.2832 | Yes |
| 14 | 352943\_1 | tph1a |  |  | 3540 | 0.921 | 0.2773 | Yes |
| 15 | 30685 | aanat2 |  |  | 3624 | 0.907 | 0.2880 | Yes |
| 16 | 30643 | acat2 |  |  | 4174 | 0.798 | 0.2825 | Yes |
| 17 | 558050 | si:ch211-127i16.2 |  |  | 4370 | 0.766 | 0.2876 | Yes |
| 18 | 30068 | cat |  |  | 4462 | 0.750 | 0.2958 | Yes |
| 19 | 100000859 | ehhadh |  |  | 4565 | 0.737 | 0.3034 | Yes |
| 20 | 492518 | haao |  |  | 5021 | 0.679 | 0.2991 | Yes |
| 21 | 100005587 | aldh9a1a.1 |  |  | 5134 | 0.663 | 0.3053 | Yes |
| 22 | 404730 | mao |  |  | 5766 | 0.610 | 0.2944 | Yes |
| 23 | 334082 | tdo2b |  |  | 5850 | 0.603 | 0.3007 | Yes |
| 24 | 100008541 | tdo2a |  |  | 5876 | 0.600 | 0.3087 | Yes |
| 25 | 557166 | acmsd |  |  | 6016 | 0.585 | 0.3129 | Yes |
| 26 | 399481 | aldh9a1b |  |  | 6718 | 0.518 | 0.2985 | No |
| 27 | 415103 | tph1b |  |  | 7790 | 0.438 | 0.2713 | No |
| 28 | 393462 | aldh2.1 |  |  | 8615 | 0.394 | 0.2512 | No |
| 29 | 100000026 | aldh3a1 |  |  | 8825 | 0.379 | 0.2502 | No |
| 30 | 793834 | hadhab |  |  | 10077 | 0.298 | 0.2153 | No |
| 31 | 100008541\_1 | tdo2a |  |  | 10281 | 0.286 | 0.2131 | No |
| 32 | 492710 | aldh16a1 |  |  | 14167 | 0.084 | 0.0923 | No |
| 33 | 570457 | aox6 |  |  | 15519 | 0.021 | 0.0501 | No |
| 34 | 791592 | ccbl1 |  |  | 15939 | 0.001 | 0.0370 | No |
| 35 | 407712\_1 | tph2 |  |  | 16433 | -0.016 | 0.0217 | No |
| 36 | 568256\_1 | asmt2 |  |  | 17277 | -0.044 | -0.0041 | No |
| 37 | 445121 | hadh |  |  | 18086 | -0.080 | -0.0284 | No |
| 38 | 557979 | aadat |  |  | 18128 | -0.082 | -0.0284 | No |
| 39 | 100150054\_1 | cyp1b1 |  |  | 18226 | -0.086 | -0.0302 | No |
| 40 | 101885014 | LOC101885014 |  |  | 18243 | -0.087 | -0.0294 | No |
| 41 | 368912 | echs1 |  |  | 18310 | -0.089 | -0.0302 | No |
| 42 | 100141344 | asmt |  |  | 18595 | -0.103 | -0.0376 | No |
| 43 | 494076\_1 | dhtkd1 |  |  | 20185 | -0.174 | -0.0850 | No |
| 44 | 406651 | ddc |  |  | 20753 | -0.201 | -0.0998 | No |
| 45 | 368242 | kmo |  |  | 21067 | -0.219 | -0.1064 | No |
| 46 | 334197 | aldh7a1 |  |  | 21094 | -0.221 | -0.1040 | No |
| 47 | 445290 | acat1 |  |  | 21467 | -0.239 | -0.1122 | No |
| 48 | 100006238 | aldh9a1a.2 |  |  | 23161 | -0.338 | -0.1604 | No |
| 49 | 399479 | dldh |  |  | 25027 | -0.448 | -0.2124 | No |
| 50 | 558050\_1 | si:ch211-127i16.2 |  |  | 26135 | -0.540 | -0.2393 | No |
| 51 | 337166 | si:dkey-40g16.6 |  |  | 26277 | -0.554 | -0.2356 | No |
| 52 | 352943 | tph1a |  |  | 26886 | -0.618 | -0.2456 | No |
| 53 | 64265 | aox5 |  |  | 27764 | -0.711 | -0.2627 | No |
| 54 | 407712 | tph2 |  |  | 27844 | -0.721 | -0.2546 | No |
| 55 | 323653 | aldh3a2a |  |  | 28024 | -0.745 | -0.2493 | No |
| 56 | 494076 | dhtkd1 |  |  | 28644 | -0.846 | -0.2563 | No |
| 57 | 550372 | afmid |  |  | 28942 | -0.907 | -0.2523 | No |
| 58 | 450003 | gcdhb |  |  | 29282 | -0.995 | -0.2483 | No |
| 59 | 100006238\_1 | aldh9a1a.2 |  |  | 29971 | -1.231 | -0.2518 | No |
| 60 | 415103\_1 | tph1b |  |  | 30140 | -1.309 | -0.2379 | No |
| 61 | 393315 | ccbl2 |  |  | 30227 | -1.353 | -0.2207 | No |
| 62 | 393860\_1 | gcdha |  |  | 30857 | -1.817 | -0.2138 | No |
| 63 | 368262 | dlst |  |  | 30863 | -1.822 | -0.1871 | No |
| 64 | 100005587\_1 | aldh9a1a.1 |  |  | 31308 | -2.447 | -0.1651 | No |
| 65 | 100536795 | wu:fb14d09 |  |  | 31372 | -2.574 | -0.1293 | No |
| 66 | 571201 | si:ch73-97h19.2 |  |  | 31873 | -4.947 | -0.0723 | No |
| 67 | 447801 | aldh8a1 |  |  | 31875 | -4.952 | 0.0005 | No |
Table: GSEA details [plain text format]

  

Fig 2: TRYPTOPHAN\_METABOLISM(DRE00380)      
 Blue-Pink O' Gram in the Space of the Analyzed GeneSet

  

Fig 3: TRYPTOPHAN\_METABOLISM(DRE00380): Random ES distribution      
 Gene set null distribution of ES for **TRYPTOPHAN\_METABOLISM(DRE00380)**
